# Changes in Myocardial Metabolism Preceding Sudden Cardiac Death

**DOI:** 10.3389/fphys.2020.00640

**Published:** 2020-06-16

**Authors:** J. Snyder, R. Zhai, A. I. Lackey, P. Y. Sato

**Affiliations:** Department of Pharmacology and Physiology, Drexel University College of Medicine, Philadelphia, PA, United States

**Keywords:** mitochondria, heart failure, sudden cardiac death, substrate utilization, lipotoxicity, glucotoxicity

## Abstract

Heart disease is widely recognized as a major cause of death worldwide and is the leading cause of mortality in the United States. Centuries of research have focused on defining mechanistic alterations that drive cardiac pathogenesis, yet sudden cardiac death (SCD) remains a common unpredictable event that claims lives in every age group. The heart supplies blood to all tissues while maintaining a constant electrical and hormonal feedback communication with other parts of the body. As such, recent research has focused on understanding how myocardial electrical and structural properties are altered by cardiac metabolism and the various signaling pathways associated with it. The importance of cardiac metabolism in maintaining myocardial function, or lack thereof, is exemplified by shifts in cardiac substrate preference during normal development and various pathological conditions. For instance, a shift from fatty acid (FA) oxidation to oxygen-sparing glycolytic energy production has been reported in many types of cardiac pathologies. Compounded by an uncoupling of glycolysis and glucose oxidation this leads to accumulation of undesirable levels of intermediate metabolites. The resulting accumulation of intermediary metabolites impacts cardiac mitochondrial function and dysregulates metabolic pathways through several mechanisms, which will be reviewed here. Importantly, reversal of metabolic maladaptation has been shown to elicit positive therapeutic effects, limiting cardiac remodeling and at least partially restoring contractile efficiency. Therein, the underlying metabolic adaptations in an array of pathological conditions as well as recently discovered downstream effects of various substrate utilization provide guidance for future therapeutic targeting. Here, we will review recent data on alterations in substrate utilization in the healthy and diseased heart, metabolic pathways governing cardiac pathogenesis, mitochondrial function in the diseased myocardium, and potential metabolism-based therapeutic interventions in disease.

## Introduction

**FIGURE 1:**
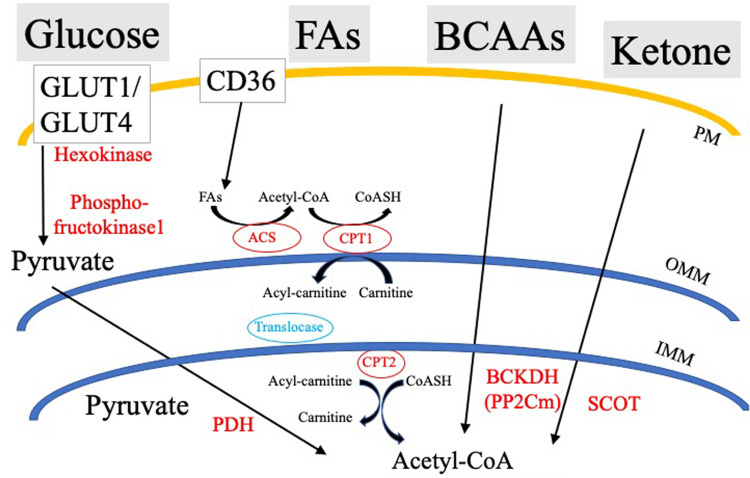
Simplified schematic of cardiac substrate utilization and substrate-specific rate-limiting/key processes. PDH, Pyruvate dehydrogenase; SCOT, CoA Transferase; BCKDH, Branched-chain α-keto acid dehydrogenase; ACS, Acyl-CoA Synthetase; OMM, Outer mitochondrial membrane; IMM, Inner mitochondrial membrane; PM, Plasma membrane; and CPT, Carnitine palmitoyltransferase.

Cardiovascular disease is the number one cause of death in the United States and worldwide, accounting for about 17.9 million deaths globally in 2015 (Benjamin et al., 2018). As the incidence of cardiac disease increases exponentially, estimates show that by 2035, expenditures related to chronic heart disease will exceed one trillion dollars (Benjamin et al., 2018). A common terminal event for cardiovascular disease is sudden cardiac arrest (SCA) leading to sudden cardiac death (SCD). SCA often results from ventricular tachyarrhythmias or ventricular fibrillation initiated by dysfunctional excitation contraction coupling. The genesis of the heartbeat is an intrinsic electrical phenomena of the heart, with electrical impulses driven by pacemaker cells at the sino-atrial node that propagate through the His-Purkinje bundle system, ultimately leading to synchronous ventricular contraction (Jalife et al., 2009). Ventricular contraction is a highly energetic process, as the heart is the most metabolically demanding organ in our body, contracting approximately 3 billion times during an average lifespan of 75 years. In spite of this extremely high energetic requirement, myocardial ATP stores are relatively low with a complete turnover of myocardial ATP pool every 10s (Neely and Morgan, 1974). To accomplish this highly bioenergetic coupling to mechanical contraction, the myocardium utilizes a gamut of circulating energetic substrates such as FAs, glucose, lactate, branched chain amino acids (BCAAs), and ketone bodies (Figure 1). These cytosolic metabolites are enzymatically processed, ultimately converging in the generation of acetyl-CoA, and/or reduced nucleotide electron carriers (FADH_2_ and NADH), that through the electron transport chain (ETC) are used to develop the proton motive force driving ATP production.

**FIGURE 2:**
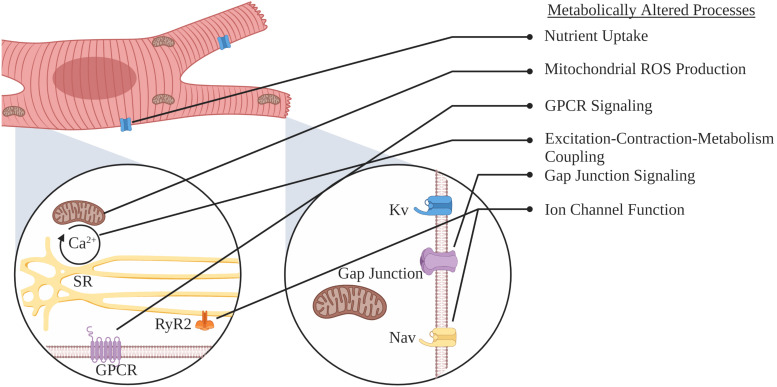
Schematic representation of myocardial adaptations preceding SCA or SCD.

While metabolic flexibility is an essential cardiac attribute, the use of each substrate is not only regulated but preference altered during development and in various cardiac pathologies. Under normal physiological conditions, energetic demand is primarily met by carbohydrates and FAs, with the latter being the major fuel source in the adult heart, where β-oxidation contributes to 60–80% of ATP production (Opie, 1968, 1969; Neely and Morgan, 1974). Nevertheless, in several cardiomyopathies, such as left ventricular hypertrophy, the adult heart reverts to a more fetal-like cardiometabolic state, switching from FA oxidation as the primary energy source to oxygen sparing glycolytic energy production (Taegtmeyer and Overturf, 1988; Depre et al., 1998). This review will focus on alterations in metabolism that occur as the heart progresses from healthy to diseased states (Figure 2). We will discuss substrate transport, the role of substrate metabolism, metabolic pathways, mitochondrial function, and ion channel modifications that precede and contribute to SCA/SCD. Lastly, we will briefly discuss potential interventions that target myocardial energy metabolism, as an approach to treat and/or prevent heart failure (HF) and SCD.

## Regulation of Glucose and Fatty Acid Entry in Cardiomyocytes

In order to meet its own bioenergetic demands, the heart expresses specialized proteins that regulate substrate entry from the blood into the cytosol of cardiomyocytes. While glucose and FAs are the major cardiac metabolic substrates, the heart can also use ketone bodies and BCAAs to meet its energetic demands (Figure 1). Transport of substrates is specific and dependent on concentration gradients and hormonal signaling. In this section, we will discuss current knowledge of various modes for glucose and FA entry into cardiomyocytes.

### Transporters of Glucose

In the adult heart, about 25 to 30% of energy used for contractile function is generated by carbohydrates (Shao and Tian, 2015). There are three known modes of glucose transport across cellular plasma membranes: (i) facilitative glucose transporters (GLUTs), which are the major form of cardiac glucose entry; (ii) sodium-glucose cotransporters (SGLTs), which use the energy of a favorable sodium gradient to transport glucose against its concentration gradient; and (iii) recently identified sugars will eventually be exported transporters (SWEETs), which promote cellular efflux of glucose (Banerjee et al., 2009; Wright, 2013). SWEETS are novel transporters of glucose that are mostly found in plants, and although understudied in humans, the human SWEET1 gene has been identified in mammary glands where it is thought to provide glucose for lactose synthesis (Chen et al., 2010). Thus far, no other major physiological function of SWEETs is known in humans.

The major path of glucose entry into the heart is via GLUTs, a family of proteins consisting of 14 members, where the main cardiac isoforms are GLUT1 and GLUT4. The expression of GLUT1 and GLUT4 is dysregulated in various cardiac diseases. In response to chronic hypoxia and ischemia, GLUT1 expression increases, leading to cardioprotection via increased glucose influx independent of insulin signaling (Brosius et al., 1997). GLUT1 is localized on the plasma membrane and plays an important role in regulating basal metabolism of cardiomyocytes. GLUT1 can be regulated by fasting and is mainly expressed in embryonic or neonatal stages of cardiac development (Kraegen et al., 1993). Genetically modified animal models have revealed an important role for GLUT1 in regulating glucose utilization for cardiac function. Murine cardiac-specific overexpression of GLUT1 enhanced cardiac glucose utilization, attenuated the development of contractile dysfunction, and improved long-term survival after transverse aortic constriction (TAC; Liao et al., 2002; Luptak et al., 2007). Moreover, acute increases in glucose uptake mediated by inducible overexpression of GLUT1 improved mitochondrial function and reduced cardiac structural remodeling but did not prevent ventricular dysfunction (Pereira et al., 2013).

Following birth, the expression of GLUT4 increases and becomes the predominant glucose transporter in adult hearts. GLUT4 is located in intracellular vesicles that translocate to the plasma membrane upon insulin stimulation or muscular contraction (Rattigan et al., 1991; Slot et al., 1991; Kraegen et al., 1993). During the development of cardiac hypertrophy, and in response to myocardial ischemia (MI), GLUT4 translocates from intracellular vesicles to the plasma membrane, increasing glucose intracellular levels (Slot et al., 1991; Young et al., 1997; Shi et al., 2016). However, GLUT4 expression is decreased in human HF with a concurrent decrease in glucose-mediated energy production (Razeghi et al., 2001; Doehner et al., 2010). Similarly, decreases in GLUT4 expression are also observed in hypertensive models showing insulin-resistance (Paternostro et al., 1995). Mouse models of GLUT4 have revealed that this transporter plays a prominent role in cardiac function. Cardiac overexpression of GLUT4 increased glycolysis but not glucose oxidation (Belke et al., 2001), while cardiac-specific deletion of GLUT4 led to cardiac hypertrophy with preserved contractile function (Abel et al., 1999). Notably, following downregulation of GLUT4, SGLT1 may mediate glucose utilization, as it is increased 2- to 3-fold in ischemic and type 2 diabetic mellitus (T2DM) hearts (Banerjee et al., 2009) and other studies have reported that SGLT1 colocalizes with GLUT1 at the sarcolemma (Turk et al., 1991; Zhou et al., 2003). Yet, the precise functional role of SGLT1 and any compensatory role in the heart remains poorly understood.

Noteworthy is the ability of incretins to promote GLUT1 expression in cardiomyocytes. In fact, incretins like glucagon-like peptide 1 (GLP-1; produced by enteroendocrine L-cells), and glucose-dependent insulinotropic polypeptide (GIP; produced by enteroendocrine K-cells; Baggio and Drucker, 2007) can regulate appetite, glucose-mediated insulin secretion from pancreatic β-cells (Varndell et al., 1985), and increase GLUT1 expression and translocation to the sarcolemma in cardiomyopathy models (Bhashyam et al., 2010). Interestingly, the impact of incretins is not limited to diabetes-related cardiac dysfunction, as GLP-1 agonists restored cardiac function in dogs with advanced dilated cardiomyopathy (DCM; Nikolaidis et al., 2004). GIP also reduces angiotensin II-induced cardiac hypertrophy and fibrosis in mice (Hiromura et al., 2016). Nonetheless, the importance of incretin signaling in metabolism and chronic disease in humans has been underscored by the association of genetic variations in GLP-1 and GIP receptors with body weight, pancreatic islet function, and risk for T2DM (Sathananthan et al., 2010; Finan et al., 2016).

### Transporters of Fatty Acids

As the healthy heart greatly depends on the oxidation of FAs for energy production, FA transport through the plasma membrane is key to myocardial metabolism (Nagoshi et al., 2011). Although FAs can passively diffuse into the plasma membrane of cells (Schaffer and Lodish, 1995), several proteins facilitate cardiomyocyte FA uptake and transport, including cluster of differentiation 36 (CD36 or FAT; Abumrad et al., 1993), plasma membrane-associated FA-binding protein or plasmalemmal FA-binding protein (FABP_*pm*_; Stremmel et al., 1985), and FA transport proteins (FATPs; Schaffer and Lodish, 1994; Gimeno et al., 2003). In addition to these membrane bound lipid transport proteins, cardiac tissue also expresses heart-FABP (HFABP or FABP3), a cytosolic lipid binding protein that binds and transports hydrophobic lipid species through the cytoplasm (Binas et al., 1999; Storch and Thumser, 2010). In the heart, CD36 is the main transporter responsible for FA uptake.

CD36 was first identified as a cellular transporter of FAs in 1993 (Abumrad et al., 1993). Several *ex vivo* and *in vivo* studies support the hypothesis that CD36 is necessary for efficient myocardial FA uptake and accumulation (Coburn et al., 2000; Bharadwaj et al., 2010). Additionally, CD36 can transduce signals that influence how cardiac FAs are utilized. For example, CD36 induces a signaling cascade that favors FA oxidation by activation of 5’ adenosine monophosphate-activated protein kinase (AMPK). CD36 forms as complex with LKB1 (AMPK Kinase) and Fyn (src kinase; Samovski et al., 2015). Binding of FA to CD36 disassociates the complex, allowing for LKB1 to phosphorylate and activate AMPK (Samovski et al., 2015). Although CD36 is thought to be the main regulator of cardiac FA transport through the plasma membrane, there does not appear to be a consensus on the role of CD36 in pathological cardiac hypertrophy (Nakamura et al., 1999). For example, in mice that were subjected to TAC, cardiomyocyte-specific ablation of CD36 resulted in a more rapid progression from compensated hypertrophy to HF (Sung et al., 2017). Conversely, in diet induced obese (DIO) mice, cardiomyocyte-specific knock down of CD36 was protective and reduced signs of pathological cardiac remodeling when compared to control mice (Zhang et al., 2015). In response to hypertrophy, the expression of FATPs are significantly decreased (Vork et al., 1992), but the physiological significance of this finding remains unclear. In order for FAs to enter the mitochondria for energy production, FAs must be activated by forming fatty-acyl-CoA. The mitochondrial enzyme that mediates FA entry, carnitine palmitoyltransferase 1 (CPT1), is located on the outer mitochondrial membrane, and modulation of its activity or expression results in alterations in FA substrate utilization in the myocardium (Opie and Knuuti, 2009). CPT1 catalyzes the transesterifcation of acetyl-CoA into acylcarnitine. Subsequently, carnitine acylcarnitine translocase (CACT) transports acylcarnitine across the inner mitochondrial membrane in exchange for a free carnitine molecule. The acylcarnitine is then reconverted into acetyl-CoA via CPT2, which is located on the inner mitochondrial membrane (Houten et al., 2016). CPT1 is the rate limiting enzyme of FA entry into the mitochondria, and its regulation is integral to the control of FA oxidation. An important post-transcriptional step in the regulation of FA oxidation involves the inhibition of CPT1 by malonyl CoA, which is formed from acetyl-CoA via acetyl-CoA carboxylase (ACC; Foster, 2012). Thus, the negative feedback inhibition of CPT1 via malonyl-CoA can avert FA-driven ATP generation, and lead to the incorporation of FA into lipid droplets in the cytosol. Excess intracellular palmitate can also lead to ceramide production and protein palmitoylation of ionic channels and mediators of cardiac conduction (Chien et al., 1996; Pei et al., 2016). Relevant to HF and SCD are the studies suggesting that reversible protein S-pamitoylation plays an important role for sodium and potassium channel biosynthesis (Schmidt and Catterall, 1987). Particularly pertinent to cardiac arrhythmias is the recent study showing that palmitoylation of the major cardiac sodium channel (Nav1.5) leads to increased channel availability and late sodium current activity, promoting the generation of arrhythmogenic events (Pei et al., 2016). Although it is currently not known if palmitoylation impacts the inward rectifier potassium channel (Ik1), there is a known reciprocity between the sodium and potassium channels at the plasma membrane that impacts excitability and arrhythmia generation (Milstein et al., 2012). Thus at the very least, the inward rectifier current could be indirectly impacted by palmitoylation of Nav1.5, this could be significant as it could also alter resting membrane potential. Palmitoylation is also known to modify the L-type calcium beta2a subunit impacting channel function (Chien et al., 1996). Thus, while it is evident that a link between cardiac metabolism and SCD exists, specific mechanisms dictating this spatiotemporal relationship remain poorly understood.

## Metabolic Pathways Governing Cardiac Function and Pathogenesis

### Metabolic Flexibility

Metabolic flexibility is a key functional aspect of a healthy heart and it relates to the ability of a cell to adapt to its environment as determined by the utilization of nutrients, oxygen, and hormonal input. It is specifically important in the heart due to the energetic demands of continuous repetitive cardiac muscle contractions and the detrimental feedback loops initiated in its absence, to be reviewed in this section. Loss of this flexibility is thought to be one of the earliest indications of cardiac dysfunction leading to HF (Harris and Das, 1991). Indeed, the failing heart has reduced bioenergetic capacity and phosphocreatine/ATP ratio (Neubauer, 2007). Glucose and FA oxidation are the two main cardiac energy sources, but chronic overreliance or overabundance of one substrate class without the corresponding bioenergetic coupling may have deleterious consequences for myocardial function (Sharma et al., 2004; Chess and Stanley, 2008). Exemplifying this metabolic-contractile link is the potential role for glucose and FA metabolism in regulating the sarcoplasmic-reticulum calcium pump (SERCA) 2a expression via SP1 (a glucose regulated transcription factor) and peroxisome proliferation-activated receptors (PPAR; a FA regulated transcription factor), respectively (Rupp and Zarain-Herzberg, 2002). Table 1 shows current models of cardiac pathology associated with SCD and their observed metabolic impact.

**TABLE 1:** SCD-disease models with associated metabolic profiles and resultant toxicity.

Disease model	Change in metabolism	Metabolic toxicity	Potential therapeutic metabolic intervention	References
TAC	↓Fatty acid oxidation ↑Glycolysis ↓BCAA Catabolism ↑Ketone Body Catabolism	Glucotoxicity Lipotoxicity	Restoration of glucose Oxidation Redirection of lipid synthesis	Shao and Tian, 2015; Muthuramu et al., 2017
I/R	↓Fatty acid oxidation ↑Glycolysis	Glucotoxicity Lipotoxicity	Restoration of FAO Restoration of glucose oxidation Redirection of lipid synthesis	Bonezzi et al., 2019; Goldenberg et al., 2019
β-Adrenergic Overstimulation	↓Fatty acid oxidation ↑Glycolysis ↓BCAA Catabolism	Glucotoxicity Lipotoxicity Insulin Resistance	Restoration of glucose oxidation Restoration of insulin sensitivity	Opie and Knuuti, 2009; Shao and Tian, 2015
Diabetes	↑BCAA/BCKA Exposure ↑Ketone Body Exposure	Lipotoxicity Insulin Resistance	↑BCAA catabolism Restoration of insulin sensitivity	Lim et al., 2020

Cardiac substrate utilization is intertwined, crossing over and impacting parallel metabolic pathways and signaling integrations. For instance, cardiac-specific deletion of ACC prevented TAC-induced decreases in FA oxidation and increased reliance on glucose (Kolwicz et al., 2012). Conversely, cardiac-specific inducible pyruvate dehydrogenase α (PDHα) deletion led to decreased glucose oxidation, decreased AMPK phosphorylation, and ischemic-induced cardiac injury (Sun W. et al., 2016). These mechanistic studies are particularly relevant to pathological conditions that involve altered substrate preferences. For example, in pathological hypertrophy and ischemic heart disease, the heart heavily relies on glucose to meet its bioenergetic needs (Eberli et al., 1991). In contrast, in T2DM cardiac glucose uptake is reduced with concurrent increase in FA oxidation enzymes leading to exacerbated cardiac hypertrophy (Paternostro et al., 1999; Domenighetti et al., 2010).

Under normal physiological conditions, more than 90% of myocardial ATP production is generated via mitochondrial oxidative phosphorylation, with the remainder being produced by anaerobic glycolysis and GTP from the tricarboxylic acid (TCA) cycle (Harris and Das, 1991). Oxidative phosphorylation in the fetal heart mainly relies on glucose, while in adult stages, there is a shift in preference toward FA metabolism. Notably, in the heart, the majority of ATP produced is quickly consumed by myofilaments during contraction. Additionally, about 25% is used to fuel cardiac sarcolemmal and sarcoplasmic reticulum (SR) ion channels and transporters (Schramm et al., 1994). The link between cardiac rhythm, myocardial work, and metabolism was first explored in the early 1900s, when notable physiologists including E. H Starling developed *in situ* models of studying these relationships (Knowlton and Starling, 1912; Patterson et al., 1914). They were able to establish relationships between preload and afterload with energetic rates, oxygen consumption, and changing blood parameters (Evans, 1939). Notably, metabolic dysfunction is ubiquitously observed in chronic heart diseases that are associated with increased risk for SCD.

### Lipotoxicity

In 1963, Philip Randle and colleagues hypothesized that the heart must utilize both glucose and FA to meet its high energy demands (Randle et al., 1963). However, successful oxidation of FA produces several factors that inhibit glycolysis, including NADH, ATP, citrate, and acetyl CoA. Thus, an environment where high glycolytic flux and FA oxidation simultaneously occur is rare. Flux through these pathways is influenced by feed/fast cycles and by exercise paradigms such that at different points, glucose and FA are used in different proportions and neither are over-accumulated (Randle et al., 1963). Overabundance of glucose or FA availability or dysregulation of the usage thereof may contribute to cytotoxic effects classified as lipotoxicity and glucotoxicity (Lundsgaard et al., 2019).

Lipotoxicity is induced by the abnormal accumulation of intra-myocellular fatty acids and lipid metabolites in non-adipose tissues such as the heart. Lipid storage in non-adipose sites contributes to altered cellular physiology and perhaps most notably insulin resistance. The summation of these detrimental effects of lipid accumulation is termed lipotoxicity (Sharma et al., 2004). Lipotoxicity can be mimicked via high fat diets that model western feeding habits and/or increased lipid transport into the myocardium (Chiu et al., 2001, 2005). However, the roles of various classes of lipid molecules in myocardial pathogenesis have only been recently explored. Increased levels of triacylglycerol (TG), the main dietary form of lipid, are associated with diseases such as obesity and T2DM. Yet, the role for TGs in inducing cellular lipotoxicity remains controversial (Drosatos and Schulze, 2013). Studies suggest that TG levels act as a marker of general lipid load, while an array of other lipid metabolites play a larger role in increasing or decreasing lipotoxicity. For example, while the role of TG in directly inducing lipotoxicity remains inconclusive, there is evidence that diacylglycerol (DG), a TG metabolite, is linked to lipotoxicity and induction of insulin resistance in muscle and liver (Inoguchi et al., 1992; Erion and Shulman, 2010). More recently, Law and colleagues have demonstrated that very long ceramide species (≥24 carbons) diminish mitochondrial function and increase apoptosis, autophagy, and mitophagy in cardiomyocytes (Law et al., 2018). Conversely, in a model where long-chain FA synthesis was re-directed toward long-chain ceramide synthesis (20–22 carbons) a cardioprotective phenotype was demonstrated in a TAC model of left ventricular hypertrophy, demonstrating the importance of lipid saturation and chain length. This latter study used a mouse model overexpressing cardiac acyl-coenzyme A synthetase 1, and thereby affected multiple lipid metabolism parameters (Goldenberg et al., 2019). However, the lipid profile following the TAC intervention specifically deviated in ceramide species accumulation (Goldenberg et al., 2019). Additionally, saturated FAs, such as palmitic acid, have been associated with increased ceramide synthesis and cardiomyocyte apoptosis when compared to unsaturated FAs, such as oleic acid (Okere et al., 2006). In fact, restoring FA oxidation in the heart by overexpression of Long-chain-FA-CoA ligase 1 (ACSL1) in a TAC model preserved cardiac function (Goldenberg et al., 2019). Together, this emphasizes the importance of lipid diversity in the heart and how they may differently impact the development of lipotoxicity.

The main source of FAs that fuel mitochondrial oxidative metabolism are circulating free FAs (FFAs) bound to albumin and/or released from TGs that are present in very-low-density lipoproteins (VLDL) or chylomicrons (Niu et al., 2004; Bharadwaj et al., 2010). As mentioned above, extracellular FAs can enter cardiomyocytes via passive diffusion, FATPs, or CD36 (Schaffer and Lodish, 1994; Gimeno et al., 2003; Bharadwaj et al., 2010). Once FFAs are in the cytosol, they are esterified to CoA via fatty acyl-CoA synthase enzymes, forming long-chain fatty acyl-CoAs (Chiu et al., 2001, 2005). These fatty acyl-CoAs can then shuttle into mitochondria via CPT1 and CPT2. CPT1 converts the long chain fatty acyl-CoA to long chain acylcarnitine in the outer mitochondrial membrane, which is then subsequently converted back to long chain fatty acyl CoA by CPT2 in the mitochondrial inner membrane (Murthy and Pande, 1984; McGarry and Brown, 1997). As mentioned previously, a major point of regulation for fatty acyl-CoA transport into the mitochondria involves CPT1, which is robustly inhibited by the presence of malonyl CoA (Foster, 2012). The levels of malonyl CoA are determined by the balance of acetyl CoA synthesis via ACC, and degradation of malonyl CoA by malonyl CoA decarboxylase (Dyck and Lopaschuk, 2002; Ussher and Lopaschuk, 2008). For example, citrate produced from the TCA cycle can move to the cytosol and activate ACC (Lane et al., 1970; Beaty and Lane, 1983), which leads to greater production of malonyl CoA and a feedback response that reduces mitochondrial FA oxidation.

During HF, fatty acyl carnitines in the cytosol and sarcolemma are accumulated up to 10 times their normal levels, particularly in cardiac regions exposed to ischemia. The relationship between lipid accumulation and electrical abnormalities has been hypothesized in the 1980s (Corr et al., 1989). Since then multiple pathways have been established by which fatty acyl carnitines affect ion channel function and therefore arrhythmogenic events. Reactive oxygen species (ROS) generated from fatty acyl carnitines interact with redox sensitive channels, such as the ryanodine receptor, thus modulating calcium release from the SR during contraction. Palmitoyl carnitine, a fatty acyl carnitine, increases the oxidation of the ryanodine receptor 2 resulting in prolonged calcium leak following opening (Roussel et al., 2015). Direct infusion of palmitoyl carnitine at levels observed in diabetic cardiomyopathy is independently arrhythmogenic in healthy animals (Roussel et al., 2015). Palmitoyl carnitine exposure to cardiomyocytes induces the slow-inactivating sodium current, impacting current inactivation thereby augmenting intracellular sodium concentration. Subsequently, palmitoyl carnitine inhibited the sodium potassium exchanger by approximately 13%, inducing an elevation in intracellular calcium (Wu and Corr, 1995). Moreover, long-chain acyl carnitines are known to reduce gap junctional conductance by 68%, a phenomenon which has been linked to the preferential accumulation of endogenous long-chain acyl carnitines at the sarcolemma during hypoxia (Wu et al., 1993). These effects alter the incidence of early after depolarizations, delayed after depolarizations, and action potential propagation promoting the development of arrhythmias.

In addition to certain lipids that may directly induce lipotoxicity, such as ceramides and DGs, ROS is generated as a byproduct of the ETC which can induce cellular stress. ROS are generated when electrons leak from the mitochondrial ETC, allowing free electrons to reduce molecular oxygen (Turrens, 2003). Certain pathological conditions, such as MI and HF, can increase ROS production via mitochondrial membrane leakage. Elevation of mitochondrial ROS can damage ETC complexes, further impairing ATP production in a failing heart (Murray et al., 2003). This leads to a vicious circle in which the electron leak increases ROS formation, and ROS formation alters the ETC to favor additional production of ROS. ROS can damage ETC complex proteins and can react with polyunsaturated FAs, which enhances the formation of peroxidized lipids that can dysregulate phospholipid membranes (Mylonas and Kouretas, 1999). In humans with diabetic cardiomyopathy and obesity-related cardiomyopathy, insulin resistance diminishes glucose uptake and utilization enhancing cardiac lipid accumulation (Boudina and Abel, 2007; Montaigne et al., 2014). As such, impairments in cardiac lipid metabolism that reduce intracellular TG-derived FA mobilization and oxidation promote an accumulation of intracellular TG (McGavock et al., 2007; O’Donnell et al., 2008). Interestingly, similar to citrate, insulin also inhibits FA oxidation through activation of ACC (Witters and Kemp, 1992). In the failing heart, insulin-induced inhibition of FA oxidation is impaired, but the effect of chronic dysregulation of genes associated with FA metabolism dominates such that TG and DG are still accumulated (Inoguchi et al., 1992; Zhang et al., 2013; Fukushima and Lopaschuk, 2016).

### Glucotoxicity

Glucotoxicity is characterized by an intracellular accumulation of glucose metabolites and subsequent impairment in glucose-mediated oxidative phosphorylation. In multiple HF etiologies, such as left ventricular hypertrophy, the heart increases its reliance on oxygen sparing glycolytic energy production. Although glucose uptake is increased, oxidative phosphorylation is diminished, or unchanged (Mori et al., 2013; Li et al., 2017a). In HF patients, augmented glycolysis is uncoupled from oxidative phosphorylation, or mitochondrial energy production, leading to the accumulation of glycolytic byproducts such as lactate and protons (Diakos et al., 2016). These glycolytic byproducts can lead to acidosis, inducing aberrations in ATPases that regulate cytosolic Na^+^ and Ca^2+^, ultimately reducing cardiac contractility (Fiolet and Baartscheer, 2000; Jaswal et al., 2011).

High glucose alone can promote pathological cardiac development by accelerating hypertrophy, promoting ER stress, increasing ROS production, and ultimately leading to apoptosis (Ng et al., 2018; Zhang X. et al., 2018; Shi et al., 2019). When uncoupled from oxidative phosphorylation, glycolysis produces glycolytic intermediates that can accelerate flux through non-anapleurotic pathways, such as the pentose phosphate pathway (PPP), hexosamine biosynthetic pathway, or lactate shuttling (Gupte et al., 2006; Gibb et al., 2017). Specifically, increased flux through the PPP occurs in both hypertrophic and chronic HF (Meerson et al., 1967; Zimmer and Peffer, 1986). Li and colleagues recently demonstrated that restoring glucose oxidation by inhibiting pyruvate dehydrogenase kinase (PDK) 4, a rate controlling enzyme of glucose oxidation, improved cardiac function, and glucose uptake post-ischemia reperfusion injury (Li et al., 2017a). Increased glucose exposure over long periods reduces BCAA catabolism, leading to an accumulation of BCAA and BCAA metabolites (Zhang X. et al., 2018). It is well established that leucine, a BCAA, is a potent activator of the mammalian target of rapamycin (mTOR) signaling pathway (Xu and Brink, 2016). Increased mTOR signaling, a known regulator of cell growth and metabolism, promotes the progression of cardiac hypertrophy and cell death (Xu and Brink, 2016). Moreover, increased glucose exposure has been shown to alter miRNAs that control protein expression and thereby regulate gross cell metabolism. For instance, elevation of miRNA-195 upregulates multiple genes associated with hypertrophic development. Exogenous administration of this oligonucleotide can promote hypertrophy and alter cardiac mitochondrial function, while the complementary oligonucleotide provides a protective effect (Shi et al., 2019; Wang et al., 2019). Lastly, increased use of glucose has been shown to induce cell growth (Kolwicz et al., 2013; Shao et al., 2018) and alter epigenetic regulation in the nucleus (Shao et al., 2018; Lombardi et al., 2019). Even though elevation in glucose appears to be an important signaling mechanism for cell growth and differentiation, specific mechanisms detailing how chronic exposure to high levels of glucose leads to glucotoxicity remains to be further characterized.

Glucotoxicity promotes protein glycation and glycosylation, which are, respectively, non-enzymatic and enzymatic protein modifications mediated by carbohydrates. These changes impact many pathways relevant to myocardial structure-functional relationships. Increased glycation drives the formation of advanced glycation end-products, which accumulate in tissues and contribute to inflammation and subsequent structural alterations, namely fibrosis (Dziubak et al., 2018). The deposition of advanced glycation end products contributes to electrical abnormalities through alterations of the myocyte cytoskeleton, altered ion channel activity, and damage to the local autonomic nervous system that contribute to regulating cardiac rhythm (Balcıoğlu and Müderrisoğlu, 2015). Advanced glycation end products specifically inhibit voltage gated K^+^ channels, Kv channels, that play a primary role in cardiac microcirculation and a secondary role in atrial repolarization, contributing to metabolic remodeling and arrhythmogenic susceptibility (Bai et al., 2015; Su et al., 2015). Relevant to atrial repolarization and His-Purkinje conduction is increased N-glycosylation of the K_2__*P*_ potassium channel, enhancing its function and transport to the cell membrane. Interestingly, while acute or chronic high glucose exposure increase N-glycosylation of the K_2__*P*_ channel, chronic exposure results in downregulation of its expression (Wiedmann et al., 2019). Acute hyperglycemia promotes the covalent modification of CaMKII by O-linked N-acetylglucosamine (O-GlcNAc) at Ser279, which activates CaMKII autonomously, leading to molecular memory as calcium concentration declines (Erickson et al., 2013). Increased O-GlcNAc-modified CaMKII, as observed in human diabetic hearts, enhances CaMKII-dependent activation of spontaneous SR calcium release, leading to augmented premature ventricular complexes that contribute to arrhythmogenicity (Erickson et al., 2013). Moreover, voltage gated sodium channels, the major ionic current responsible for ventricular membrane depolarization, are also heavily modulated by glucose-based and other posttranslational modifications that can be dysregulated by metabolism. For instance, regulation of Nav1.5 glycosylation and sialyltransferase activity impacts the sodium voltage-gated activity and propensity for arrhythmias (Ufret-Vincenty et al., 2001; Ednie et al., 2013). Moreover, exposure of high glucose in CHO cells transiently overexpressing the human Nav1.5 channel led to a rightward shift in voltage dependence of conductance and steady-state fast inactivation, which was attributed to the observed increase in ROS (Fouda et al., 2020). Whether this mechanism is also pertinent to cardiac cells remains to be determined.

### Ketone Body Metabolism

In HF, cardiac substrate utilization of glucose and FAs decrease with a concurrent increased reliance on ketone bodies and BCAAs (Kolwicz et al., 2013; Aubert et al., 2016; Bedi et al., 2016). In the hypertrophied and failing heart, ketone body metabolism supplements energy production following decreased FA oxidation (Aubert et al., 2016). Ketones are primarily produced within hepatocytes and form acetyl-CoA via 3-oxoacid-CoA transferase (SCOT) in extrahepatic tissue to generate energy (Laffel, 1999). The major determinant of cardiac ketone oxidation rates is circulating ketone levels (Laffel, 1999; Ikegami et al., 2017). In human HF, ketone utilization plays an important role in maintaining cardiac metabolism (Aubert et al., 2016; Bedi et al., 2016). Recently, Schugar and colleagues reported that mice subjected to TAC with a cardiomyocyte-specific ablation of SCOT (the rate limiting enzyme in ketone oxidation) displayed increased mitochondrial dysfunction, and accelerated pathological cardiac remodeling (Schugar et al., 2014). Additionally, overexpression of cardiac D-β-hydroxybutyrate dehydrogenase 1 (BDH1) decreased ROS and apoptosis in mice subjected to TAC (Uchihashi et al., 2017). Studies in isolated rat cardiomyocytes exposed to simulated hypoxia showed that β-hydroxybutyrate increases contraction and calcium in a dose-dependent manner (Klos et al., 2019). Experiments in isolated working hearts subjected to 4-weeks of TAC showed that enhancing ketone body oxidation increased energy production although it did not show significantly improvement in cardiac efficiency (Ho et al., 2019). Thus, increased myocardial ketone oxidation may be an adaptive mechanism for the failing heart. These results from animal models are supported by recent findings in humans with chronic HF that show decreased expression of lipid utilization proteins with increased expression of ketone catabolic genes (Aubert et al., 2016; Bedi et al., 2016).

Recently, the “ketogenic diet” has become popular, yet it remains unknown whether enhancing long-term ketone oxidation is adaptive or maladaptive to the human heart. There is concern about the common use of this diet, especially in obese and diabetic individuals with an increased risk for SCA/SCD. Currently, it appears that the benefit of weight loss associated with this diet may outweigh potential adverse cardiac metabolic changes (Harvey et al., 2019). In wild-type mice, ketogenic diets increase ketone oxidation genes, decrease gene profiles associated with glucose utilization, and do not alter expression of FA utilization genes (Shimizu et al., 2018). Nevertheless, long-term effects of increasing ketone bioenergetics remain to be fully detailed.

### BCAA Metabolism

Branched chain amino acids can be metabolized in the cardiac muscle via the branched-chain α-ketoacid dehydrogenase (BCKDH) complex (Neinast et al., 2019). Alterations in cardiac BCAA metabolism are associated with cardiac pathologies. For example, defective BCAA catabolism disrupts glucose signaling and sensitizes the heart to ischemic-reperfusion injury (Li et al., 2017b). The expression of BCAA catabolic enzymes is decreased in HF patients, in conjunction with increased levels of BCAA and branched chain keto acids (Sun H. et al., 2016). In fact, studies in TAC-induced mice suggest that increasing BCAA metabolism preserves cardiac structure and function (Chen et al., 2019). BCAA supplementation and more generally high protein diets are common practices in order to obtain desired physiological outcomes such as in resistance training. In a large observational study, it appears that both low (<1 *g*/kg body weight) and high protein (>1.38 *g*/kg body weight) intake associate with increased risk for cardiovascular events as well as all-cause mortality even when accounting for risk factors and renal function (Halbesma et al., 2009). Specific conclusions regarding the BCAA element of these dietary classifications, however, require further studies. BCAAs only represent a small part of total protein intake, and high protein diets in observational studies will most likely differ in macronutrient and micronutrient makeup. BCAA supplementation in addition to resistance exercise training in late-stage HF patients does not improve physical and functional capacities (Pineda-Juarez et al., 2016). Nevertheless, multiple clinical trials continue to investigate a potential interaction between BCAA metabolism, BCAA supplementation, and exercise in different types of HF (Halbesma et al., 2009; Pineda-Juarez et al., 2016).

## Pathological Mitochondrial Function

### Mitochondrial Calcium Handling

Deterioration in contractile mechanics of a heart that is hypertrophied, dilated, or fibrotic correlates with altered excitation-contraction coupling and failing bioenergetics (Franz et al., 1992; Nishimura et al., 2006). Contractile force generation requires calcium signaling; while intracellular SR calcium is reviewed in detail elsewhere (Eisner et al., 2020), in this section, we will specifically provide a brief overview of mitochondrial calcium handling. Mitochondrial calcium dynamics are determined by ATP/ADP ratio, transporter expression levels, and other regulatory signals (Zhou and Tian, 2018). Calcium entry in the mitochondrial inner membrane can be facilitated by the mitochondrial calcium uniporter (MCU). Systemic MCU knockout studies did not reveal any major baseline phenotype although mitochondrial calcium handling was diminished (Pan et al., 2013). Cardiac-specific MCU deletion led to improved cardiac function post-IR (Luongo et al., 2015), supporting the notion that MCU does not impact baseline physiology but instead modulates energetic upregulation by increasing contractility or sympathetic stress signaling. Moreover, MICU1, a regulator of the MCU (Mallilankaraman et al., 2012), was recently shown to possess an additional function where it controls cristae junction and anchoring of the MCU complex (Gottschalk et al., 2019). Mitochondrial calcium extrusion, however, is dependent on mitochondrial Na/Ca exchanger (NCLX). Murine models of inducible cardiac-specific NCLX knockout increased arrhythmogenicity and SCD, while overexpression of NCLX was cardioprotective post-IR (Luongo et al., 2017). Calcium extrusion can also be elicited via the mitochondrial permeability transition pore (MPTP), which promotes apoptotic signaling. Although the molecular identity of the MPTP is debated and unknown, recent studies have suggested that dimerization of the F1F0 ATP synthase is responsible for the pore forming MPTP (Urbani et al., 2019), with a prominent role for the c-subunit of the ATPase in MPTP formation (Neginskaya et al., 2019). Most importantly, recent studies have linked the relevance of mitochondrial calcium dynamics to human cardiac pathology. In diabetic rats, cardiac mitochondrial MCU expression is decreased, diminishing mitochondrial calcium and subsequently blunting pyruvate dehydrogenase activity, which it is the rate-limiting process in glucose oxidation (Suarez et al., 2018). In agreement are studies showing decreased levels of MCU and MICU1 in cardiac tissue from ischemic HF patients (Luongo et al., 2015). The feasibility and specificity of pharmacologically targeting these mitochondrial channels and signaling pathways remain to be determined.

### Mitochondrial ROS Generation

ROS are generated when electrons leak from the mitochondrial ETC, allowing free electrons to reduce molecular oxygen (Turrens, 2003). Certain pathological conditions, such as MI and HF, increase ROS production via mitochondrial membrane leakage. Elevation of mitochondrial ROS damage ETC complexes further impairing ATP production in the failing heart (Murray et al., 2003; Redout et al., 2007; Dubouchaud et al., 2018). ROS generation is in concert with previously discussed alterations in mitochondrial calcium dynamics and reduced oxidative phosphorylation (Ide et al., 1999; Zhang H. et al., 2018). Dysfunction of complex 1, 2, and 3 of the ETC have been associated with ROS production (Redout et al., 2007; Dubouchaud et al., 2018). ROS can also trigger the reversible, transient, and minimally concerted opening of the MPTP, which is thought to have a housekeeping physiological function of releasing ROS to the cytosol. Prolonged ROS generation triggers MPTP opening, which leads to ROS-induced-ROS release promoting the destruction of the mitochondrion that may be propagated inter-mitochondrially, culminating in cell death (Zorov et al., 2000). Interestingly, deletion of cyclophilin-D, a regulator of the MPTP, led to resistance to MPTP opening and improved cell survival post-IR (Baines et al., 2005). Additionally, loss of cyclophilin-D led to greater hypertrophy and HF (Elrod et al., 2010). Mitochondrial oxygen levels can be sensed by the mitochondrially localized NADPH-oxidase 4 (Nox4). Unlike other Nox family members, Nox4 is constitutively active where 90% of the electron flux through isolated Nox4 produces H_2_O_2_ and 10% forms superoxide (Nisimoto et al., 2014). Cardiac-specific Nox4 overexpression is detrimental to cardiac function post-ischemia-reperfusion injury (Ago et al., 2010), re-enforcing the crucial role that Nox4 plays in regulating myocyte function. An extensive review of mitochondrial ROS is found elsewhere (Cadenas, 2018).

There is a strong link between ROS and cardiac ion channel dysfunction directly impacting cardiac electrophysiology, contractile deficiencies, and SCD. For instance, the ryanodine receptor, can be oxidized by ROS resulting in altered calcium release (Roussel et al., 2015). The carotid body is a group of neurosensory cells that sense blood oxygenation and inform ventilation and adrenergic stimulation to increase blood flow in response to ischemia. Voltage-gated potassium channels (Kv) channels are significantly impacted by redox-based reactions in the coronary vasculature and the carotid body. As a result of altered metabolism there is an increase in ROS in the coronary vasculature and carotid body, resulting in greater oxygenation and perfusion rates in an attempt to reduce hypoxic exposure (Rogers et al., 2006; Moreno-Dominguez et al., 2020). Therein ROS, plays an important direct role in the hyperemic response to increased cardiac output. Yet, long-term activation of this mechanism, either due to metabolic or structural remodeling, has adverse consequences as ROS alters a multitude of vital cardiac pathways that promote SCD. Although Kv channels are also found elsewhere in the heart, including the sino-atrial node and His-Purkinje system, how ROS alters action potential propagation in these specialized cardiac cells are currently unknown. Importantly, Kv channels have varying subunit composition in various tissues as well as modulation by other cellular redox agents such as NADH and NADPH which increase the activity of Kv channels (Dwenger et al., 2018). Kv channel activation via ROS in an acutely adaptive mechanism, such as increasing oxygenation and perfusion, contrasts Kv channel inhibition by glycation and products discussed in the section “Glucotoxicity” (Su et al., 2015; Moreno-Dominguez et al., 2020). While it is reasonable to conclude that metabolic toxicity would dysregulate this family of ion channels, further studies are required to delineate how ROS modification of various ionic channels are integrated in the resultant arrhythmic incidence of the heart.

## Past and On Going Therapeutic Interventions to Improve Glucose and FA Metabolism

### Clinical Approaches to Modulate Glucose Metabolism

The interaction between insulin resistance and diabetic cardiomyopathy has been a major driver for unraveling therapies that enhance glucose metabolism. Enhancing glucose uptake has been attempted by insulin infusion called glucose–insulin–potassium (GIK) therapy, which involved exposure to high concentrations of insulin during acute ischemia (Sodi-Pallares et al., 1962). Some studies have suggested that GIK infusion during ischemia reduced infarct size and improved post-ischemic cardiac dysfunction, but clinical trials were inconsistent and a meticulous glucose control was essential for those patients (Van den Berghe et al., 2003; Malmberg et al., 2005). GLP1 can increase glucose uptake by increasing GLUT2 expression in pancreatic islets (Villanueva-Penacarrillo et al., 2001). Exenatide is a human GLP1 receptor agonist that is currently being investigated as a potential adjunct therapy for HF. A study conducted in 2007 showed that patients treated with exenatide had decreased mortality from cardiovascular causes when compared to patients treated with placebo or standard of care (Erdmann et al., 2007). Exenatide can also contribute to vasorelaxation due to the effect on opening K_*ATP*_ channels and therefore preventing vessel dysfunction (Selley et al., 2014). Conversely, recent studies showed no significant benefit on cardiovascular events in T2DM patients (Mentz et al., 2018). Liraglutide is an FDA-approved drug that is currently used to treat T2DM patients. Liraglutide also activates the GLP1 receptor, and is currently being investigated as a potential treatment for HF. T2DM patients treated with liraglutide have improved mortality rates compared to placebo treated patients (Cohen and Beckey, 2016). Circulating GLP1 is quickly metabolized by dipeptidyl peptidase (DPP)-IV, and thus, a DPP-IV inhibitors can be used as adjuvant with GLP1 agonists to improve GLP1 efficacy (Deacon, 2004). One study demonstrated that an inhibitor of DPP-IV, sitagliptin, improves left ventricular function in patients with coronary artery disease (Read et al., 2010). It has been suggested that the mechanism for the cardioprotective effect of sitagliptin is via upregulation of the transient receptor potential channel (TRP), therefore increasing Ca^2+^ influx (Al-Awar et al., 2018). In contrast, a recent study revealed no benefit on reducing HF risk in patients with diabetes treated with sitagliptin (Nauck et al., 2019).

Pyruvate is a key glycolytic metabolite which has positive inotropic actions in both healthy and dysfunctional hearts, improving left ventricular function (Mentzer et al., 1989). Pyruvate can potentiate the inotropic response of β-adrenergic receptor compounds and result in increased intracellular Ca^2+^ transients via improvement of SERCA in failing human myocardium (Hermann et al., 2002). The pyruvate dehydrogenase complex (PDC) metabolizes pyruvate to acetyl-CoA, and PDK inactivates this complex via phosphorylation, leading to a buildup of glucose metabolites. Inhibiting PDK with dichloroacetate (DCA) is one approach that increases the utilization of glucose in the heart (Stacpoole et al., 1998). Clinical evidence has shown that DCA can weakly improve cardiac function through the regulation of voltage-gated K^+^ channels, but unfortunately, this compound has a major toxicity risk for the liver, kidney, and nervous system (Michelakis et al., 2002). Other alternative compounds currently under development for PDK inhibition have also shown substantial side effects in multiple organs (Morrell et al., 2003; Roche and Hiromasa, 2007). Currently, another effective modulator of PDK, PS10, is being evaluated as potential drug target (Wu et al., 2018). Moreover, SGLT2s are expressed in the early proximal tubule of the kidney (Vallon et al., 2011), and while SGLT2 inhibitors have been developed with the intent to improve glucose control in diabetic patients via its impact on renal sodium-glucose re-absorptive function, it has sparked enthusiasm for the treatment of HF. The EMPA-trial revealed that T2DM patients treated with empagliflozin possessed a 38% relative risk reduction in death from cardiovascular causes (Zinman et al., 2015). Empagliflozin also significantly regulates calcium and sodium exchange, which can be a potential mechanism for the cardioprotective effect (Lee et al., 2019). While there is a consensus that improving glucose metabolism is an attractive pharmacological intervention, specific, safe, and effective compounds that provide cardiovascular benefits still need to be developed.

### Therapeutic Strategies Targeting Fatty Acid Utilization

As mitochondrial FA utilization is dependent on CPT-1, this transporter is an attractive pharmacological target for HF, such that inhibiting FA oxidation can result in parallel increase of glucose utilization (Xu et al., 1995). Perhexiline maleate was developed as an antianginal drug in the 1970s (Ashrafian et al., 2007). It potentially inhibits the uptake of fatty-acyl-CoA via mitochondrial CPT-1 (Murray et al., 2005), and this inhibition of FA metabolism improves left ventricular ejection fraction (LVEF) in patients with chronic HF (Lee et al., 2005). Perhexiline also potentially protects the heart from cardiac dysfunction by inhibiting Human ether-a-go-go-related gene (HERG) channels (Walker et al., 1999). Etomoxir is an irreversible inhibitor of CPT-1 (Lopaschuk et al., 1988) that also increased SERCA2a expression, improving SR Ca^2+^ handling and cardiac function (Vetter and Rupp, 1994). It was first developed and tested in clinical trials in 1984, passing phase I and II, and becoming a promising drug for HF patients (Schmidt-Schweda and Holubarsch, 2000). Unfortunately, etomoxir treatment resulted in abnormally high liver transaminase levels and now is only used as an experimental tool for inhibiting FA oxidation (Holubarsch et al., 2007).

Trimetazidine is an inhibitor of 3-ketoacyl CoA thiolase, the terminal enzyme of FA β-oxidation (Kantor et al., 2000; Lopaschuk et al., 2003). Trimetazidine treatment reduced FA oxidation and induced a compensatory increase in glucose oxidation. Trimetazidine also reduced the accumulation of lactic acid caused by the buildup of fatty acids. Further, this drug modified calcium dynamics by inhibiting SERCA activity and modifying the density of Ca^2+^ channels in cardiomyocytes impacting left ventricular systolic and diastolic function (Kiyosue et al., 1986; Banach et al., 2006; Meng et al., 2006). In clinical trials trimetazidine significantly increased LVEF (Vitale et al., 2004), myocardial perfusion, oxidative metabolism, and work efficiency (Fragasso et al., 2006), and is being investigated as a potential therapeutic strategy for HF patients (Dezsi, 2016).

Ranolazine was FDA-approved in 2006 for the treatment of stable angina pectoris (Reddy et al., 2010). Ranolazine was first thought to inhibit FA metabolism similarly to trimetazidine, but the drug concentration required to inhibit FA β-oxidation was much higher than the recommended therapeutic dose, indicating an alternative mechanism of action (McCormack et al., 1996). Studies suggest that the primary mechanism of action for ranolazine is via inhibition of the late sodium current and diastolic Ca^2+^overload, which benefit patients with diastolic dysfunction (Antzelevitch et al., 2004; Belardinelli et al., 2006; Fraser et al., 2006). Ranolazine has been shown in clinical studies to have therapeutic efficacy for many kinds of cardiomyopathies (Sossalla et al., 2008; Maier et al., 2013).

Lastly, PPARs are key transcriptional factors that regulate FA uptake and metabolism, and modulation of various PPAR family members may have a beneficial impact for HF. The expression of PPARα is decreased during TAC-induced HF (Kaimoto et al., 2017). However, ischemic failing rats hearts treated with the PPARα agonist fenofibrate did not show improvements in left ventricular function (Morgan et al., 2006). Additionally, fenofibrate treatment may lead to left ventricular dysfunction in mice with hypertension (Ogata et al., 2004; Brigadeau et al., 2007). Post-trial follow up from the ACCORD study confirmed the original overall neutral results of the study but revealed a beneficial impact of fenofibrate therapy in reducing cardiovascular diseases in a specific group of participants with diabetes, hypertriglyceridemia, and low high-density lipoprotein cholesterol (Elam et al., 2017). PPARγ agonists are currently used in the treatment of T2DM with suggested indirect benefits to cardiovascular disease (Elam et al., 2011). Table 2 summarizes previous and ongoing therapeutic interventions for glucose and FA metabolism.

**TABLE 2:** Therapeutic interventions that may regulate glucose and FA metabolism.

	Target	Name	Clinical use	Arrhythmia events	References
Glucose metabolism	GLP-1 agonist	Exenatide	Under investigation-No significant benefit	Yes, unknown ionic alteration	Mentz et al., 2018
		Liraglutide	Under investigation	No	Cohen and Beckey, 2016
	DPP-IV inhibitor	Sitagliptin	Under investigation-No significant benefit	No	Read et al., 2010; Nauck et al., 2019
	SGLT2 inhibitor	Empaglifloxin	Glucose management-Cardiac benefit under investigation	Antiarrhythmic effect via regulates Na^+^-Ca^2+^ exchanger	Zinman et al., 2015
	PDK inhibitor	Dichloroacetate (DCA)	Toxicity	No	Stacpoole et al., 1998
		PS10	Under investigation	Not yet determined	Wu et al., 2018
Fatty acid metabolism	CPT-1 inhibitor	Etomoxir	Adverse effects	Antiarrhythmic effect via improving Ca^2+^ handling	Vetter and Rupp, 1994; Holubarsch et al., 2007
		Perhexiline maleate	Under investigation	Antiarrhythmic effect via inhibit HERG channels	Lee et al., 2005
	β-oxidation inhibitor	Trimetazidine	Under investigation	Antiarrhythmic effect via modify Ca^2+^ channels	Fragasso et al., 2006; Dezsi, 2016
		Ranolazine	Under investigation	Antiarrhythmic effect via regulate both Na^+^ and Ca^2+^	Sossalla et al., 2008; Maier et al., 2013
	PPARα agonist	Fenofibrate	Under investigation	Antiarrhythmic effect via K^+^ channel	Morgan et al., 2006; Elam et al., 2011, 2017

## Conclusion

Atrial fibrillation, tachyarrhythmias, and dysfunction in mechanical junctions preceding cardiac structural remodeling and fibrosis may all contribute to SCD (Jalife et al., 2009; Sato et al., 2009, 2011, 2018). More recently, cardiac metabolism has been studied as a potential early causal mode that promotes and supports SCD (Wu et al., 1993; Roussel et al., 2015). Metabolic flexibility and shifts in metabolic utilization are major determinants of adaptive and maladaptive cardiac signaling. This is particularly evident in T2DM patients, where aberrant glucose control is thought to contribute to cardiac dysfunction in more than 50% of patients (Dunlay et al., 2019). While metabolic pathways are somewhat plastic, detangling specific substrate utilization pathways has been particularly challenging because substrate preference alterations include horizontal crosstalk of various metabolic pathways. Undoubtedly, intervening at early maladaptive stages is key to delay or impede the development of HF and SCD. Detailing which enzymatic reactions are critical and pharmacologically targetable is essential to developing specific compounds that intervene accurately and efficaciously in the fight against SCD events. Future studies will be paramount in unraveling key enzymatic events for the development of novel compounds and determining which population groups are more prone to be responsive to these interventions.

## Author Contributions

JS and RZ contributed equally to this work. JS, RZ, AL, and PS reviewed the literature, drafted the manuscript, and critically revised the manuscript.

## Conflict of Interest

The authors declare that the research was conducted in the absence of any commercial or financial relationships that could be construed as a potential conflict of interest.
